# Preparation, Characterization, and Antioxidant Capacity of Xanthone–Urea Complex

**DOI:** 10.3390/ma18112658

**Published:** 2025-06-05

**Authors:** Catherine Ortega, Manami Nomura, Mizuki Ohtomo, Florencio Arce, Gerard Lee See, Yutaka Inoue

**Affiliations:** 1 Pharmaceutical Research and Drug Development Laboratories, Department of Pharmacy, School of Health Care Professions, University of San Carlos, Cebu 6000, Philippines; 2 Faculty of Pharmacy and Pharmaceutical Sciences, Josai University, 1-1 Keyakidai, Sakado 3500295, Saitama, Japan; 3 Pharmaceutical Sciences Division, National Research Council of the Philippines, Taguig City 1631, Philippines

**Keywords:** Xanthone, urea, drug complexation, solubility enhancement, antioxidant capacity

## Abstract

Xanthones are a group of polyphenolic compounds widely known to have antitumor, anti-inflammatory, antibacterial, antifungal, antiviral, and antioxidant properties. To fully utilize their therapeutic potential, this study aimed to enhance the solubility of a poorly soluble xanthone by preparing a 1:1 molar ratio of xanthone–urea complex utilizing a cogrinding method via a vibration rod mill. DSC analysis revealed the disappearance of the characteristic endothermic peaks of xanthone (177 °C) and urea (136 °C) in the ground mixture (GM), along with the appearance of a new endothermic peak at 185 °C, indicating potential complexation. Additionally, new peaks were observed in the PXRD patterns of the GM at 9.1°, 12.0°, 14.0°, 18.6°, 19.6°, and 24.6°, suggesting structural changes that were also observed in SEM morphology. FTIR spectroscopy revealed significant shifts in the -NH and C=O peaks of xanthone and urea, as well as the disappearance of a -CN peak. Altered diffusion coefficients for both xanthone and urea were measured using DOSY-NMR, accompanied by notable improvements in solubility and dissolution profiles. The GM exhibited nearly a 2-fold increase in solubility, reaching 88.08 ± 1.25 µg/mL at 24 h and 90.97 ± 0.98 µg/mL at 72 h, alongside a 2-fold and 5-fold increase in dissolution at 0.21 µg/mL and 0.51 µg/mL for the physical mixture (PM) and GM, respectively. Furthermore, an enhanced antioxidant capacity was observed, as demonstrated in the calculated Trolox equivalent (TE) value, which increased from 1.48 ± 1.12 for xanthone alone to 1.65 ± 1.03 in the xanthone–urea complex. These findings confirm the successful complexation of xanthone and urea in a 1:1 molar ratio.

## 1. Introduction

**Figure 1:**
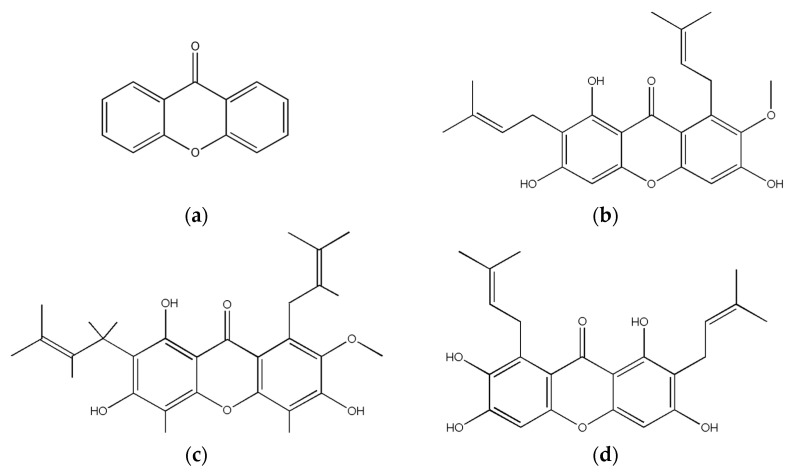
Chemical structure of xanthone and commonly studied derivatives (**a**) xanthone core, (**b**) α- mangostin, (**c**) β-mangostin and (**d**) γ-mangostin.

Xanthones are a group of polyphenolic, naturally occurring compounds commonly sourced from the pericarp of *Garcinia mangostana*, also known as mangosteen, an indigenous plant in Southeast Asia [1]. The most studied xanthones (Figure 1) are α-, β-, and γ-mangostins, garcinone E, 8-deoxygartanin, and gartanin [2]. The tricyclic xanthone core, which is present in these compounds, is critical for electron delocalization, which confers antioxidant and radical scavenging properties. These derivatives have also been reported to have antitumor, anti-inflammatory, antibacterial, antifungal, and antiviral properties [1,3,4,5,6]. Amongst these, xanthones are commercially used as a dietary supplement due to their potent antioxidant capacity [7,8]. Four different mechanisms of xanthone antioxidant activity have been reported from studies on mangosteen: free radical scavenging, chelating ability, reducing power, and lipid oxidation inhibitory ability [9]. Through these mechanisms, xanthones demonstrate promising applications in the prevention and treatment of diseases such as cancer, diabetes mellitus, skin inflammation, Alzheimer’s and Parkinson’s disease, in cosmeceuticals as an antiaging ingredient, and other degenerative conditions linked to various oxidative pathways [1,10,11,12,13,14].

Despite their notable antioxidant properties, mangosteen xanthones face significant pharmacokinetic issues due to their poor solubility and limited bioavailability [15]. They are metabolized in the intestinal cells via conversion to phase II metabolites, with absorption dependent on the assembly and secretion of chylomicrons, favoring absorption when taken with other products of lipid digestion [15]. The estimated water solubility of xanthone at 25 °C is 4.5 mg/L with a log Kow of 3.39. Based on the United States Pharmacopeia (USP), solubility at this value is classified as practically insoluble, indicating very low solubility and low absorption. Reportedly, the oral bioavailability of mangosteen xanthone dissolved in an aqueous solution in rodents was estimated to be as low as 0.4% [16]. This makes its utilization in the body a challenge, especially in the development of suitable drug formulations.

Complexation is a technique widely used in drug formulation studies to enhance the solubility of poorly water-soluble drugs. Various methods of complexation, such as coprecipitation, solvent evaporation, and spray drying, have been utilized for this purpose. While these methods are effective in producing uniform and stable drug complexes, they necessitate the use of organic solvents, which not only raises concerns regarding toxicity, but also results in significant solvent waste during processing. Mechanochemical methods, such as ball milling and rod milling, offer solvent-free processing and scalability [17,18,19].

Complexing agents such as cyclodextrins, nicotinamide, phospholipids, and urea have also been studied as solubility enhancers [17,18,19,20]. Urea is a hydrotropic agent with the ability to increase the water solubility of nonpolar compounds by disrupting the water clusters surrounding the nonpolar molecule, causing an increase in entropy, and allowing solubilization [21]. Its hexagonal crystal structure is reported to improve solubility by enclosing the compound molecules in its pores [22]. With this property, urea has been used to enhance the solubility of poorly water-soluble drugs such as ellagic acid, with a 4-fold increase; ibuprofen and ascorbyl palmitate, with a 3.5-fold increase; carbamazepine and nicotinamide up to a ~30-fold increase; and diclofenac [22,23,24,25]. Notably, the antioxidant activities of ellagic acid and ascorbyl palmitate were both retained upon complexation, with the antioxidant property of the ellagic acid–urea complex showing higher antioxidant capacity.

This study aimed to formulate a xanthone–urea complex prepared using a vibration rod mill. Vibration milling is frequently employed in pharmaceuticals to attain particle sizes in the micron and sub-micron range, which are essential for formulating various dosage forms and drug delivery systems such as drug nanoparticles and nanosuspensions. The vibration rod mill method effectively increases the surface area of drug particles in a grinding process, which generates heat due to the mechanochemical effect produced, allowing complex formation without the use of organic solvents [16,26]. This reduction in particle size, in addition to complexation with urea, will synergistically improve the water-solubility and antioxidant capacity of xanthones. Further exploration of this process, along with the ability of urea as a solubility enhancer via drug complexation, would allow a seminal approach to its application in other poorly soluble pharmaceutical agents.

## 2. Materials and Methods

### 2.1. Materials

The active ingredient xanthone (XA) was procured from Fujifilm Wako (Lot No. SKG5178, Fujifilm Wako Pure Chemical Corporation, Osaka, Japan). 2,2′-Azobis (2-amidinopropane) dihydrochloride (AAPH) was purchased from Fujifilm Wako (Lot No. CKL4958, Fujifilm Wako Pure Chemical Corporation Osaka, Japan), while 6-hydroxy-2,5,7,8-tetramethylchroman-2-carboxylic acid (Trolox^®^) was purchased from Tokyo Chemical Industry (Lot No. O2K4I-LC; SACJO-DJ, Tokyo, Japan).

### 2.2. Methods

#### 2.2.1. Complex Preparation

The physical mixture (PM) was prepared by mixing a 1:1 molar ratio of xanthone (100 mg) and urea (30.61 mg) using a vortex mixer (Scientific Industries, New York, NY, USA) for 1 min. The PM is then ground for 60 min by a vibration rod mill to produce the ground mixture (GM).

#### 2.2.2. Differential Scanning Calorimetry

Differential Scanning Calorimetry (DSC) measurements were performed using a differential scanning calorimeter (Differential scanning calorimeter, Hitachi High-Tech Corporation, Tokyo, Japan). About 5 mg of the sample was carefully deposited into a sealed chromated aluminum pan. The temperature was gradually increased from 20 °C to 300 °C at a constant rate of 10 °C per minute, while ensuring a continuous flow of nitrogen gas at a rate of 100 mL/min, 14–20 psi.

#### 2.2.3. Powder X-Ray Diffraction

Powder X-ray Diffraction (PXRD) measurements were performed using the MiniFlex II PXRD system (Powder X-ray diffractometer, Rigaku, Tokyo, Japan). The diffraction intensity was measured using a NaI scintillation counter (Labtron Equipment, Surrey, UK). PXRD was performed using Cu Kα radiation (30 kV, 15 mA), with a scan rate of 4°/min and a scan range of 5–40° (2θ). The powder sample was placed evenly on the top flat surface of the glass plates.

#### 2.2.4. Fourier Transform Infrared Spectra

The Fourier Transform Infrared (FTIR) spectra were obtained from an FT/IR-460 Plus (FTIR spectrophotometer, JASCO, Tokyo, Japan) with samples prepared via the potassium bromide (KBr) pellet method with an integration frequency of 32 times, a resolution of 4 cm^−1^, and a wavenumber range of 4000–650 cm^−1^. The pellets were prepared by mixing the sample with KBr at a ratio of 1/10 and by manual pressing. Background correction was performed using KBr alone.

#### 2.2.5. Scanning Electron Microscopy

Particle size and shape were measured using a scanning electron microscope S3000N SEM (Scanning electron microscope, Hitachi High-Tech Corporation, Tokyo, Japan) at an accelerating voltage of 15 kV. The vacuum gold-steaming time of the samples was 70 s.

#### 2.2.6. Solubility Test

A solubility test was performed to determine the solubility of the XA/UR complex in laboratory-reagent grade distilled water. A concentration of 200 μM each of the XA, PM (XA/UR = 1/1), and GM samples were added individually to 10 mL of distilled water and mixed using a magnetic stirrer (AS One, Tokyo, Japan) at 25 °C and 100 rpm for 1, 3, 6, 24, and 72 h (n = 3). The test solution was then filtered through a 0.45 μm nylon membrane filter, diluted with 9 mL methanol, and quantified for its drug content using a GENESYS 10S UV-Visible spectrophotometer (UV-Vis spectrophotometer; Thermo Fisher; Madison, WI, USA).

#### 2.2.7. Dissolution Test

The dissolution test was performed using NTR-593 dissolution apparatus (Dissolution apparatus, Toyama Sangyo Co., Ltd., Tokyo, Japan) according to the JP XVII revised dissolution test using the paddle method. A sample (equivalent to 50 mg XA) was placed in 500 mL distilled water at 37 ± 0.5 °C and stirred at 100 rpm. Aliquots (5 mL) of dissolved samples were collected at 5, 10, 15, 30, and 60 min and filtered through a 0.2 μm nylon membrane filter (n = 3). After sample collection, the same amount of distilled water was added at the same temperature to maintain a constant volume of the dissolution medium. The dissolution rates were analyzed using high performance liquid chromatography (HPLC; Waters Alliance, Milford, MA, USA).

#### 2.2.8. DOSY-NMR

Diffusion-ordered NMR spectroscopy analysis was performed using a Varian 700 MHz NMR spectrometer (NMR spectrometer, Agilent Technologies, Santa Clara, CA, USA) with an HCN probe operating at 699.7 mHz and DMSO-d6 as solvent. Other conditions were as follows: mixing time, 100 ms; waiting time, 1 s; integration frequency, 256; and a temperature of 25 °C.

#### 2.2.9. Oxygen Radical Absorbance Capacity

An oxygen radical absorbance capacity (ORAC) assay was performed to assess whether the improved solubility and dissolution characteristics of the formed complex contributed to enhancing its antioxidant capacity. Both the XA, UR, PM, and GM samples were subjected to this assay with Trolox (TX; Vitamin E) as a reference compound (n = 3). 2,2′-azobis(2-methylpropionamidine) dihydrochloride (AAPH) (203.4 mg) was dissolved in 10 mL of 75 mM phosphate buffer (pH 7.2). A fluorescein sodium stock solution (4 µM) is made in 75 mM phosphate buffer and stored in an amber bottle at 4 °C. Prior to use, the stock solution is diluted to 50 nM with 75 mM phosphate buffer. A total of 150 µL of the diluted fluorescein sodium stock solution was added to all wells of a 96-well microplate. Blank wells received 25 µL of 75 mM, while standards received 25 µL of Trolox dilution, and samples received 25 µL of the sample. The plate was then allowed to equilibrate by incubating for a minimum of 30 min at 37 °C. The reaction was initiated by the addition of 25 µL of AAPH solution for a final volume of 200 µL. The fluorescence was then monitored kinetically with data taken every minute using the ClarioStar microplate reader (Microplate reader, BMG Labtech GmbH, Ortenberg, Germany).

Excitation was performed at 485 nm with a 20 nm bandpass, and emission was measured at 528 nm with a 20 nm bandpass. Initiation of reaction is followed by shaking at maximum intensity for 10 s. Fluorescence of each well was then measured kinetically every 60 s using the auto scale option. The ORAC values were calculated using the following equations:

(1) $$AUC=\left(R1/R1\right)+\left(R2/R1\right)+\left(R3/R1\right)+\cdots +\left(Rn/R1\right)$$

where *R*1 is the fluorescence reading at the initiation of the reaction, and *Rn* is the last measurement. Trolox equivalents (*TE*) will be calculated from the linear regression slope of the positive control and sample compound to determine antioxidant capacity using the equation:

(2) TE=mCompound/mTrolox

#### 2.2.10. Statistical Analysis

The data generated from characterization tests and assays of the XA, PM, and GM were expressed as the mean + standard deviation (SD). One-way analysis of variance (ANOVA) and Tukey’s post hoc analysis were conducted to compare the differences between the results from the analysis of XA, PM, and GM samples, with the statistical significance set at *p* < α = 0.01. GraphPad Prism 5.03 (GraphPad Prism version 5.03, GraphPad Software Inc., San Diego, CA, USA; GPW5-079309-NCH-2077; accessed 9 November 2024) was utilized for data analysis.

## 3. Results

### 3.1. Physical Appearance

**Figure 2:**
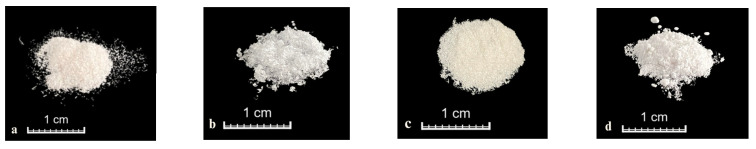
Photo images of (**a**) xanthone (XA), (**b**) urea (UR), (**c**) physical mixture (PM), (**d**) ground mixture (GM) (XA/UR 1:1).

The pure xanthone powder (a) appears as a fine, slightly compact powder with an off-white coloration and granulated, uniformly sized particles. Urea (b), on the other hand, has a crystalline appearance with larger particle size, smoother surface, and a more elongated shape. In comparison, the physical mixture (c) shows a smaller, granulated mixture with off-white coloration, while the ground mixture (d) appears as a fine, white solid powder with fluffy, loose aggregates (Figure 2).

### 3.2. DSC Measurement

**Figure 3:**
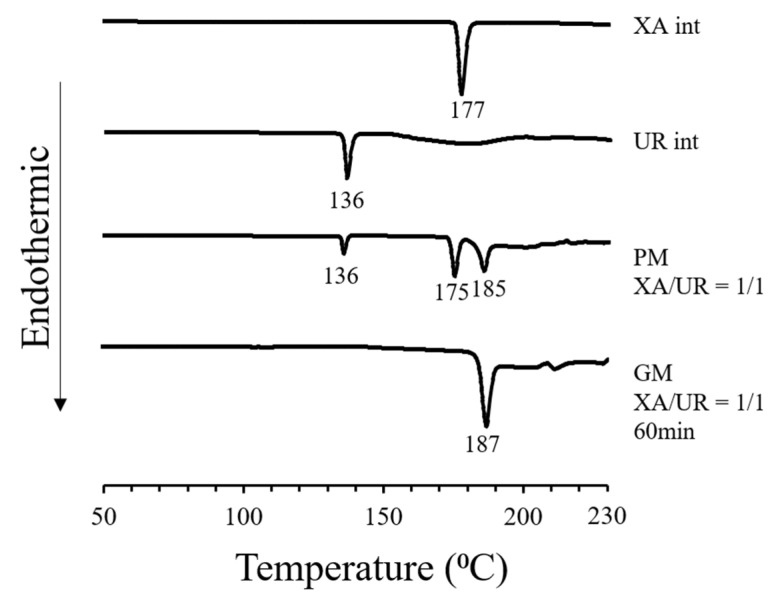
DSC curves of xanthone (XA), urea (UR), physical mixture (PM), and ground mixture (GM).

Differential scanning calorimetry was performed to view the changes in thermal behavior and confirm complex formation (Figure 3).

For pure xanthone, an endothermic peak due to melting was observed at around 177 °C. For pure urea, an endothermic peak due to melting was observed at around 136 °C. For the physical mixture, the endothermic peaks for both xanthone urea were still present, followed by an endothermic peak potentially due to the complex formed, observed at around 185 °C for both PM and GM. In comparison to the mixed ground product, the endothermic peaks of xanthone and urea disappeared, and only the endothermic peak thought to be due to the complex was observed. Additional endothermic peaks are found at 240 °C for PM and 260 °C for GM, which may be attributed to the systems’ degradation products upon exposure to high temperatures.

### 3.3. PXRD Measurements

**Figure 4:**
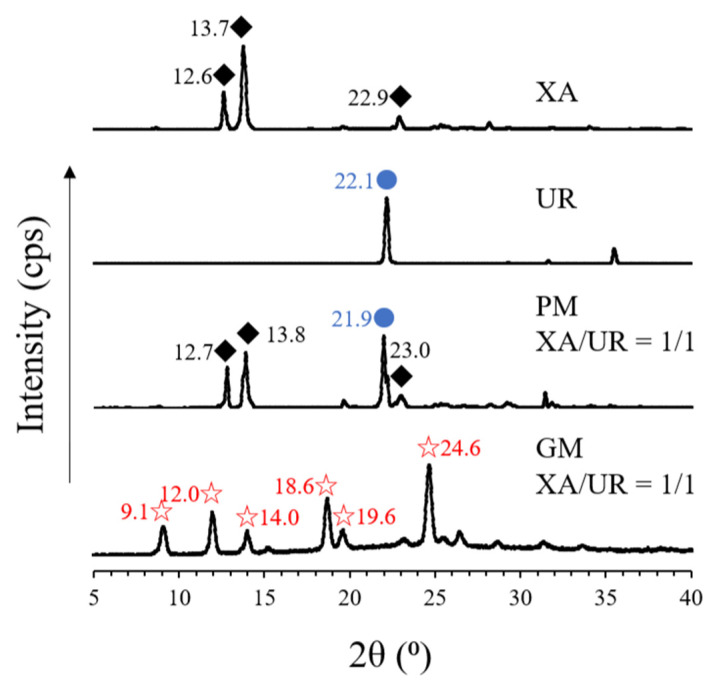
PXRD patterns of pure xanthone (XA) and urea (UR), physical mixture (PM), and ground mixture (GM). The diamond, blue dots, and star shapes indicate similar peaks that appear across the different samples.

PXRD analysis was performed to observe changes in the crystal structure after cogrinding (Figure 4).

In XA, characteristic peaks (2ϴ) were observed at 12.6°, 13.7°, and 22.9°, while in UR, a characteristic diffraction peak was observed at 22.1°. The derived peaks from both XA and UR are also visible in PM, which are observed at 12.7°, 13.8°, and 23.0° for XA and 21.9° for UR. This contrasts with the observed peaks on the GM, where characteristic diffraction peaks for both XA and UR disappeared and new peaks appeared at 9.1°, 12.0°, 14.0°, 18.6°, 19.6°, and 24.6°, respectively.

### 3.4. FTIR Absorption Spectrum

**Figure 5:**
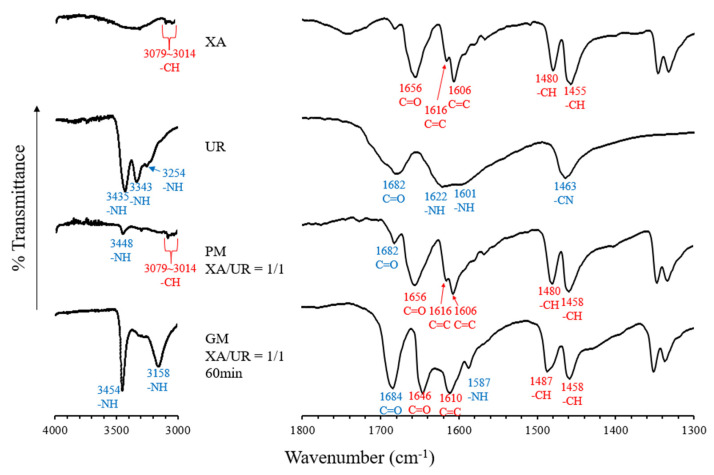
FTIR absorption spectrum of pure xanthone (XA) and urea (UR), physical mixture (PM) and ground mixture (GM).

FT-IR spectroscopy was used to investigate the intermolecular interactions within the XA and UR complex (Figure 5). In the spectrum of XA, a peak corresponding to the -CH group was observed at 3079–3014 cm^−1^, a C=O peak was observed at 1656, C=C peaks were found in 1616 and 1606 cm^−1^, and -CH peaks were recorded at 1480 and 1455 cm^−1^. For UR, peaks related to the -NH group appeared at 3448, 3433, 3254, as well as in 1622 and 1601 cm^−1^. Peaks attributed to the C=O appeared in 1682 cm^−1^ and -CN at 1463 cm^−1^. When XA and UR were mixed in a 1:1 ratio in the physical mixture (PM), peaks at 3079–3014 cm^−1^ (-CH group) and 3448 cm^−1^ (-NH group) were still present, indicating minimal interaction between the two components in this state. XA-derived peaks for C=O, C=C, and -CH were also still present, along with a C=O-derived peak from UR. In comparison, the ground mixture (GM, XA/UR = 1/1, 60 min) showed significant shifts, including the appearance of new peaks at 3158 and 1587 cm^−1^, which can be attributed to the -NH group, suggesting stronger interactions. Additionally, the C=O peak of UR at 1682 cm^−1^ shifted to 1684 cm^−1^, and the C=O peak of XA also moved from 1656 to 1646. Moreover, a shift in the -CH peaks from 1480 to 1487 cm^−1^ was observed, along with the disappearance of the -CN peak at 1463 cm^−1^ in the GM.

### 3.5. SEM Analysis

**Figure 6:**
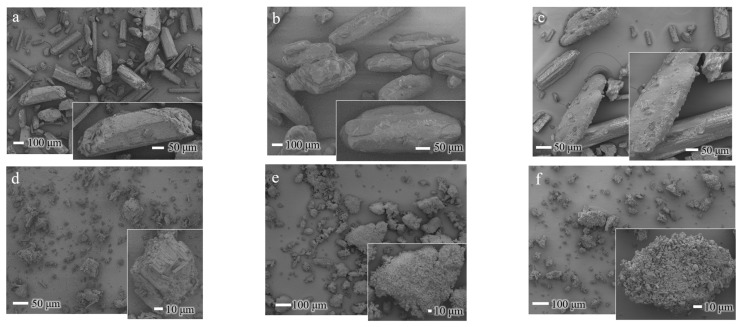
SEM images of XA/UR systems (**a**) xanthone (XA), (**b**) urea (UR), (**c**) XA/UR physical mixture (PM) (**d**) Ground XA (**e**) Ground UR (**f**) XA/UR ground mixture (GM).

The SEM images (Figure 6) illustrate the morphological characteristics of XA and UR in their pure, physically mixed (PM), and ground states, as well as the ground mixture (GM) of XA/UR.

In pure XA, the particles exhibit a distinct crystalline structure with a well-defined, rod-like shape with sharp edges, which is true to the nature of XA crystals. With intact UR, the particles display irregular, elongated block-like structures with relatively smooth surfaces, which is a characteristic of crystalline urea. For both ground XA and UR, fragmented particles with irregular, smaller sizes and rougher surfaces are observed, with noticeable small aggregates formed. Meanwhile, the GM shows a highly irregular, agglomerated morphology with significant blending of the components, as observed in the loss of distinct crystalline structures of each component.

### 3.6. DOSY-NMR Measurement

**Figure 7:**
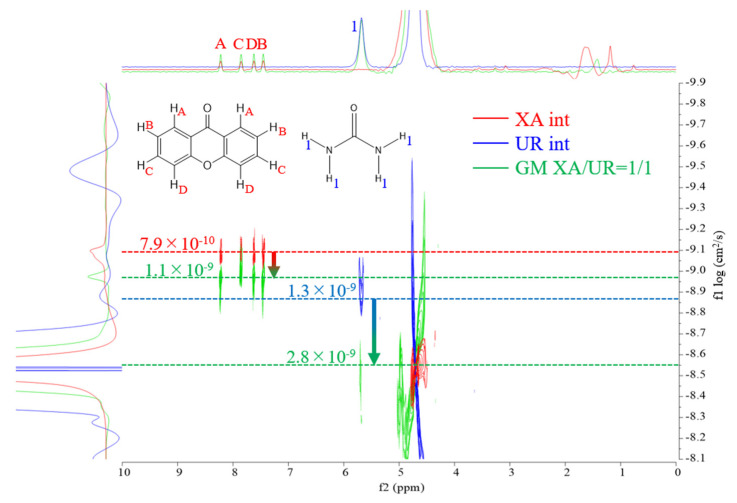
DOSY–NMR spectra of XA (red), UR (blue), and GM (green).

DOSY (diffusion-ordered spectroscopy) NMR was used to evaluate the molecular diffusion behavior of XA, UR, and their ground mixture (GM, XA/UR = 1/1), as shown in Figure 7.

Self-diffusion coefficients were used to analyze the intermolecular interactions between the components. As reflected in Table 1, in the individual spectrum of XA, the diffusion coefficient was observed at 7.9 × 10^−10^ cm^2^/s, indicating the characteristic mobility of XA in solution. For UR, the diffusion coefficient was measured at 1.3 × 10^−9^ cm^2^/s, reflecting its smaller molecular size and faster diffusion compared to XA. In the GM spectrum, the diffusion coefficient significantly shifted to 1.1 × 10^−9^ cm^2^/s for XA and 2.8 × 10^−9^ cm^2^/s for UR, suggesting the formation of a new species or complex with altered molecular mobility.

### 3.7. Solubility Test

**Figure 8:**
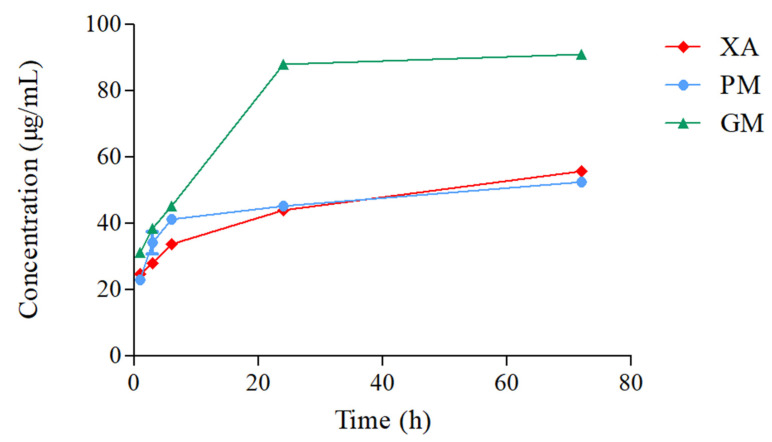
Solubility profiles of XA/UR systems in distilled water at 25 °C (n = 3).

The solubility of xanthone was recorded at 1, 3, 6, 24, and 72 h (Figure 8). For XA, mean solubility was recorded in an increasing manner from 24.70 ± 0.31 µg/mL at 1 h, 28.14 µg/mL at 3 h, 33.92 ± 0.16 µg/mL at 6 h, 43.94 ± 0.56 µg/mL at 24 h, and 55.76 ± 0.95 µg/mL at 72 h. Similar results were recorded for PM, with a solubility of 22.99 µg/mL at 1 h, 34.28 ± 3.36 µg/mL at 3 h, 41.32 ± 1.22 µg/mL at 6 h, 45.29 ± 0.41 µg/mL at 24 h, and 52.42 ± 0.41 µg/mL at 72 h. With GM, a slight increase in solubility was recorded at 31.30 ± 1.28 µg/mL for 1 h, 38.61 ± 0.31 µg/mL for 3 h, and 45.29 ± 0.27 µg/mL for 6 h. Noticeably, an almost 2-fold increase in solubility at 88.08 ± 1.25 µg/mL and 90.97 ± 0.97 µg/mL was documented at 24 h and 72 h, respectively. A one-way analysis of variance (ANOVA) was conducted to compare the solubility of XA, PM, and GM across the different time points. The results indicate a significant difference between the solubility of XA and PM vs. GM at the 24 h and 72 h mark (*p* < 0.0001).

### 3.8. Dissolution Test

**Figure 9:**
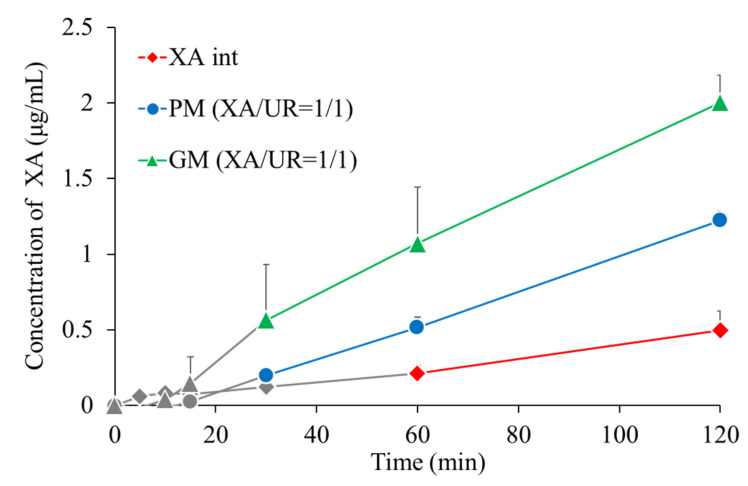
Dissolution profiles of XA/UR systems (n = 3).

Dissolution tests were conducted to confirm changes in dissolution properties (Figure 9). The gray areas in the figure indicate samples in which the xanthone concentration was below the quantification or detection limit. At 60 min, the xanthone concentration of xanthone alone was 0.21 μg/mL, that of PM was 0.51 μg/mL, and that of GM was 1.1 μg/mL. The xanthone concentrations of PM and GM were approximately 2.4 and 5.2 times higher, respectively, than that of xanthone alone.

### 3.9. Oxygen Radical Absorbance Capacity

**Figure 10:**
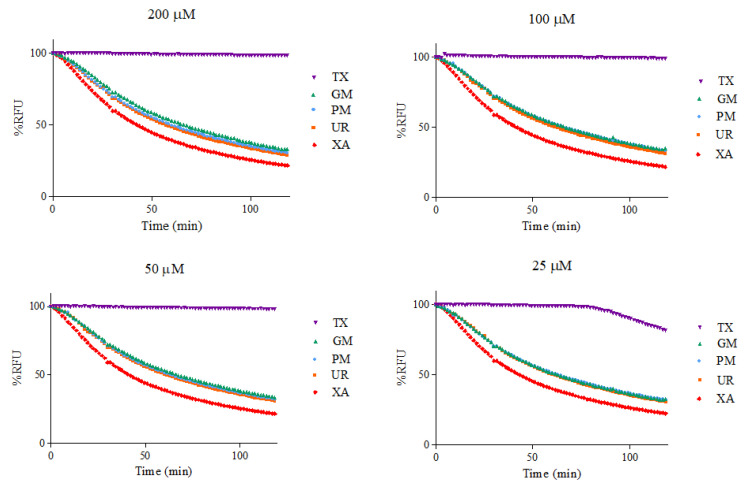
Fluorescence intensity by %RFU at different sample concentrations.

**Figure 11:**
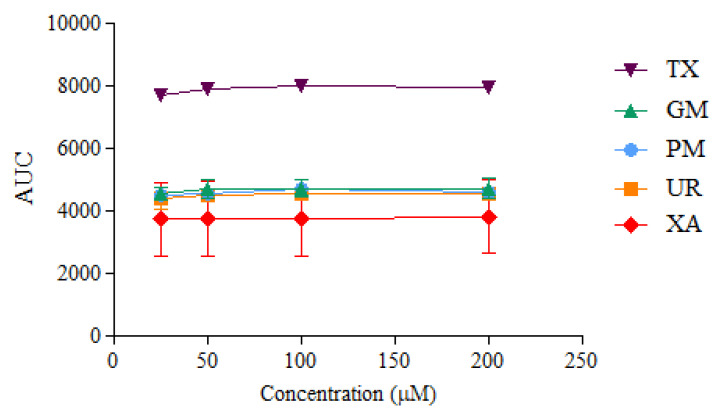
AUC values calculated at different sample concentrations (n = 3).

ORAC kinetic curves of the xanthone (XA), urea (UR), XA-UR physical mixture (PM), XA-UR ground mixture (GM), and Trolox (TX) were recorded over 2 h, with each measurement read every 90 s (80 cycles) (Figure 10). The AUC values were calculated for each sample and plotted against the respective concentrations (Figure 11).

Among the five samples assayed, XA recorded the lowest AUC values across all concentrations. There is no significant difference between the AUC of XA, UR, PM, and GM (*p* > 0.01), indicating near-similar antioxidant capacities. The positive control, Trolox, had the highest AUC values. The linear regression slope for each sample was used to calculate Trolox equivalents (TE) for each compound, with GM observed to have the highest value at 1.6542 ± 1.03 in comparison to XA alone at 1.4895 ± 1.12, followed by UR at 1.5635 ± 1.18. Notably, a higher TE was also observed for UR than XA (Table 2).

## 4. Discussion

The therapeutic potential of xanthone is limited by its poor aqueous solubility. To overcome this challenge, a xanthone–urea (1:1 XA/UR) drug complex was successfully formulated using a cogrinding method via vibration rod mill. Complexation was methodically confirmed through various characterization tests including observations from the samples’ differential scanning calorimetry (DSC) thermograms, powder X-ray diffraction (PXRD) patterns, and Fourier transformed infrared (FTIR) spectra along with evaluation of the compounds’ behavior in aqueous solution using diffusion ordered spectroscopy (DOSY), solubility testing, dissolution testing and measuring its antioxidant capacity.

In the DSC thermogram, the appearance of new and distinct endothermic curves for the ground mixture (GM) was observed, which indicates significant physicochemical changes in the compound, potentially due to complexation and the formation of new molecular interactions between xanthone and urea. The same observations have been reported in urea complexation with ellagic acid and ascorbyl palmitate [22,24]. This is further supported by the FTIR absorption spectra, which reveal shifts in the peak positions of the GM, implying the formation of the hydrogen bonds or other intermolecular interactions between the -NH group of UR and the C=C and C=O groups of XA. The new interactions identified in FTIR correspond to the altered thermal behavior observed in DSC, confirming that the components are no longer in their original crystalline forms but are interacting in a new, distinct phase. Similar interactions have also been reported in other hydrogen bond donors like thiourea complexation with carbamazepine and guanidine with curcumin [26,27]. It is noticeable that the characteristic XA and GM peaks are still present in the PM thermogram, indicating stronger molecular interactions within the GM compared to the PM. Physical changes in the crystalline structure, as observed in the disappearance of the characteristic diffraction peaks of both XA and UR in the PXRD patterns of the GM, along with the appearance of new diffraction peaks, also substantiate the formation of a complex between the two compounds. The SEM images also show a significant morphological transformation in the GM, with the disappearance of individual compounds’ defined crystalline structures, reduced particle size, well-combined, and uniformly shaped particles. This reduction in particle size increased the particle surface area, providing more areas for the aqueous solvent and the XA-UR complex to interact with each other.

To evaluate the effect of complexation on xanthone behavior in aqueous solutions, DOSY-NMR, solubility, and dissolution tests were performed. A shift in the diffusion coefficients was measured from the DOSY-NMR spectra of the GM, suggesting the formation of a new species with altered molecular mobility. The observed shift indicates a reduction in the individual mobility of XA and UR due to interactions, likely through hydrogen bonding or other intermolecular forces. The overlapping of signals in the GM spectrum also provides additional evidence of strong interactions between XA and UR in the mixture. The formation of hydrogen bonds, as exhibited in the FTIR results between xanthone and urea, is expected to enhance its solubility. Notably, the GM exhibits a nearly two-fold improvement in both solubility and dissolution profiles, as detailed in the findings. The observed increase in solubility is attributed to the formation of the complex, which leads to enhanced hydrotropic properties and the formation of additional hydrogen bonds between xanthone and urea. Improvement in solubility is critical for enhancing the bioavailability of xanthones in the body, as it promotes more efficient dissolution in aqueous biological fluids, allowing better absorption.

The differences observed in the results of the characterization tests between the PM and GM show that heat, as part of the cogrinding process, may be an important factor in the formation of the complex and the subsequent molecular interactions involved. Grinding disrupts the crystalline structure of both xanthone and urea, increasing their amorphous content. This higher amorphous content enhances molecular mobility, enabling stronger hydrogen bonding between the functional groups (-NH, C=O).

Xanthone is widely used for its antioxidant properties. The study hypothesized that while xanthone donates hydrogen atoms from its aromatic rings to neutralize free radicals, urea forms hydrogen bonds and acts as a hydrogen bond acceptor via its carbonyl oxygen. The simultaneous activities of XA and UR as a single compound are expected to produce a synergistic effect, resulting in more effective neutralization of reactive oxygen species (ROS) and a broader spectrum of protection against oxidative-stress-related conditions such as inflammation, neurodegeneration, and cardiovascular diseases. However, the ORAC assay reveals that although complexation with urea produces an increase in antioxidant capacity, this increase is not attributable to the enhanced solubility but rather to the intrinsic antioxidant properties of urea alone. Urea and its derivatives have been proved to have antioxidant properties [28,29,30]. Based on the results of the assay, urea had a higher antioxidant capacity than xanthone, and combining both drugs in a physical and ground mixture increased this antioxidant capacity. However, the calculated AUC values indicate that increasing sample concentrations does not substantially enhance antioxidant capacity. This limitation may be attributed to the poor solubility of the compounds in the aqueous phosphate buffer used for the ORAC assay [31].

While previous studies on xanthone complexes have primarily focused on co-crystallization, spray-drying, and coacervation techniques, this study demonstrates the potential of cogrinding as a simpler and solvent-free method to enhance xanthone solubility in aqueous solvents [32,33,34]. Although the increase in solubility is lower in comparison with the almost 40-fold solubility increase observed with other techniques [32,33], the solvent-free preparation method employed in this study aligns with green chemistry principles, making it a promising approach for sustainable drug development. However, although this study demonstrates the potential of the xanthone–urea complex, the exact molecular interactions require further investigation through advanced techniques such as solid-state NMR or molecular modeling. Additionally, in vitro and, further, in vivo studies, such as diffusion cell systems (e.g., Franz diffusion cells) or ultrafiltration through membranes, are needed to validate the biological relevance of the enhanced solubility and antioxidant activity observed in vitro.

## 5. Conclusions

The successful preparation of a xanthone–urea complex through vibration rod milling as a cogrinding method has been confirmed by various characterization tests that evaluated its physicochemical properties. The complexation of XA/UR in the GM (XA/UR = 1/1) significantly enhanced the solubility and dissolution of XA. Moreover, an increase in antioxidant capacity was noted in the xanthone–urea complex compared to the individual components. This demonstrates the potential of urea as a functional excipient and vibration rod milling as a scalable, solvent-free method for developing novel complex formulations. Looking forward, this formulation approach could also be applied to other poorly soluble drugs. The enhanced solubility of the xanthone–urea not only improves its suitability for oral administration but also creates opportunities for its development as a therapeutic agent in inflammation and other oxidative stress-related disorders.

## Figures and Tables

**Table 1:** Self-diffusion coefficients of XA, UR, and GM in solution.

Self-Diffusion Coefficient (cm^2^/s)		
	XA	UR
Intact	7.9 × 10^−10^ ± 0.04	1.3 × 10^−9^ ± 0.06
GM	1.1 × 10^−9^ ± 0.03	2.8 × 10^−9^ ± 0.01

**Table 2:** Calculated ORAC Trolox equivalents (TE) of sample compounds.

Compound	Trolox Equivalent
XA	1.4895 + 1.12 TE
UR	1.5635 ± 1.18 TE
PM	1.3976 ± 0.84 TE
GM	1.6542 ± 1.03 TE

## Data Availability

The original contributions presented in this study are included in the article. Further inquiries can be directed to the corresponding authors.

## Abbreviations

The following abbreviations are used in this manuscript:

XA	Xanthone
UR	Urea
PM	Physical Mixture
GM	Ground Mixture
DSC	Differential Scanning Calorimetry
FTIR	Fourier Transform Infrared Spectroscopy
PXRD	Powder X-ray Diffraction
DOSY	Diffusion Ordered Spectroscopy
ORAC	Oxygen Radical Absorbance Capacity
RFU	Relative Fluorescence Units
